# Impaired Focal Adhesion Kinase-Grb2 Interaction during Elevated Activity in Hippocampal Neurons

**DOI:** 10.3390/ijms160715659

**Published:** 2015-07-10

**Authors:** Sachiko Murase

**Affiliations:** 1 Laboratory of Molecular Biology, National Institute of Neurological Disorder and Stroke, National Institutes of Health, Bethesda, MD 20892, USA; 2 Department of Biology and Neuroscience and Cognitive Sciences Program, University of Maryland, College Park, MD 20742, USA; E-Mail: smurase@umd.edu; Tel.: +1-301-405-7222

**Keywords:** focal adhesion kinase (FAK), Grb2, Erk1/2, signal transducer and activator of transcription 3 (STAT3), integrin β1, matrix-metalloproteinase (MMP), excitatory/inhibitory (E/I) balance, survival signaling

## Abstract

Excitatory/inhibitory imbalances are implicated in many neurological disorders. Previously, we showed that chronically elevated network activity induces vulnerability in neurons due to loss of signal transducer and activator of transcription 3 (STAT3) signaling in response to the impairment of the serine/threonine kinase, extracellular-signal-regulated kinases 1/2 (Erk1/2) activation. However, how phosphorylation of Erk1/2 decreases during elevated neuronal activity was unknown. Here I show the pErk1/2 decrease induced by 4-aminopyridine (4-AP), an A-type potassium channel inhibitor can be blocked by a broad-spectrum matrix-metalloproteinase (MMP) inhibitor, FN-439. Surface expression levels of integrin β1 dramatically decrease when neurons are challenged by chronically elevated activity, which is reversed by FN-439. Treatment with 4-AP induces degradation of focal adhesion kinase (FAK), the mediator of integrin signaling. As a result, interactions between FAK and growth factor receptor-bound protein 2 (Grb2), the adaptor protein that mediates Erk1/2 activation by integrin, are severely impaired. Together, these data suggest the loss of integrin signaling during elevated activity causes vulnerability in neurons.

## 1. Introduction

It is critical to keep a neuron’s balance of excitatory/inhibitory (E/I) inputs within a certain range to maintain normal and healthy brain function. Although neurons can adjust their excitability in response to global changes in input levels [1,2], E/I balance is compromised in many neurological disorders, in which neurons experience chronically elevated activity [3,4,5,6]. In diseases characterized by altered E/I balance, neurons face degeneration of axons and dendrites and even death [7,8,9].

Previously, we showed that elevated neuroactivity impairs phosphorylation of signal transducer and activator of transcription 3 (STAT3) at Ser-727 [10]. The phosphorylation of STAT3 was dependent on the activation of the serine/threonine kinase, Erk1/2. When Erk1/2-STAT3 signaling was impaired, these neurons became neurotrophin-dependent for their survival. STAT3 signaling was necessary and sufficient for neurons to become neurotrophin independent. Neurons challenged by chronic elevation of activity showed increased expression of the tumor suppressor p53 and its pro-apoptotic target gene product, Bax. These results suggest that neurons become vulnerable during the elevated neuroactivity. However, the mechanism by which Erk1/2 activity is impaired during elevated activity is unknown.

Integrins consist of heterodimers (α and β) [11], and serve as receptors for extracellular matrix (ECM) proteins that send intracellular signals such as Erk1/2 that are critical for cell survival [12,13]. Integrins mediate cell-ECM adhesion, which recruits FAK to participate in focal adhesion [14]. FAK then interacts with the adaptor protein Grb2 and mediates Erk1/2 activation through integrin signaling [15]. Therefore, changes in integrin signaling may regulate the activation of Erk1/2 and its target, STAT3.

Matrix-metalloproteinases (MMPs) are extracellular zinc-dependent endopeptidases that regulate cell-ECM interactions through the cleavage of ECM proteins [12,13]. Subtypes of MMPs are expressed in neurons and activated by neuroactivity under both normal [16,17,18] and pathological conditions [19,20,21]. These results raise the possibility that integrin signaling may be altered in neurons challenged by elevated neuroactivity.

Here I show inhibition of MMPs can effectively block the impairment of Erk1/2 and its target, STAT3 activation. Chronic elevation of neuroactivity reduces surface integrin β1 levels, leading to calpain-dependent FAK degradation. This may be a common mechanism for inducing vulnerability in neurons in neurological disorders that are implicated in altered E/I balance.

## 2. Results

**Figure 1:**
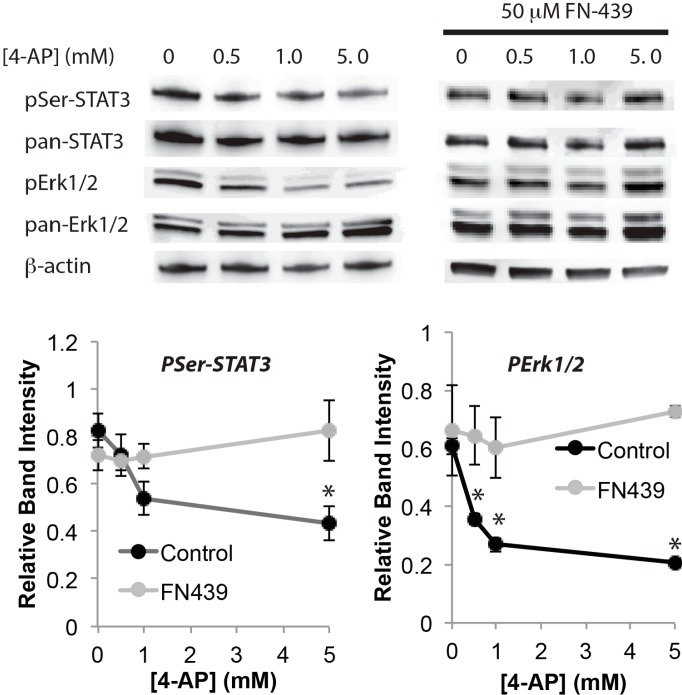
Inhibition of matrix-metalloproteinases (MMPs) blocks the decline of levels of survival signaling induced by chronic network activation. Western blot analysis of total STAT3 (pan-STAT3), pSer-STAT3, total Erk1/2 (pan-Erk1/2) and pErk1/2. β-Actin was used as control (*n* = 4, * *p* < 0.05, one-way ANOVA). Neurons were treated with the indicated concentrations of 4-AP for 18 h with or without 50 μM FN-439.

Chronic elevation of network activity may induce pathological activation of MMPs [22], and we previously showed that high MMP activity corresponded to neuronal vulnerability [23]. Therefore, the effect of a broad-spectrum MMP inhibitor, FN-439 was tested on a key survival signaling pathway that is impaired by chronically elevated neuroactivity. When dissociated cultured neurons were incubated overnight with A-type potassium channel blocker 4-AP, total Erk1/2 increased whereas total STAT3 remained unchanged (Figure 1). Levels of pSer-STAT3 and pErk1/2 decreased in a manner dependent on 4-AP concentration (Figure 1) as reported previously [10]. However, when the neurons were incubated together with the MMP inhibitor FN-439, both pSer-STAT3 and pErk1/2 were insensitive to 4-AP (Figure 1).

**Figure 2:**
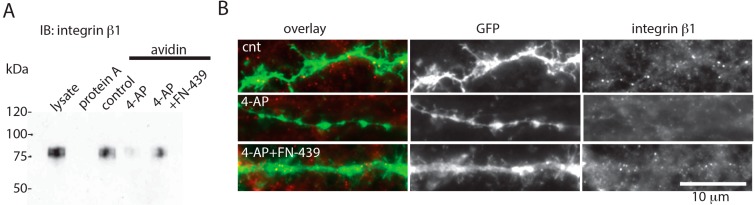
Surface expression of integrin β1 decreases when neuronal activity is elevated. (**A**) Cell surface biotin-labeling analysis. Western blot analysis of integrin β1. Lysate and protein A-sepharose (instead of avidin-conjugated sepharose) are used as positive and negative controls, respectively; and (**B**) Immunostaining against integrin β1 in non-permeabilized GFP-expressing neurons. Neurons were treated for 18 h with 5 mM 4-AP with or without 50 μM FN-439.

Next, the effect of MMP activity on integrin signaling was investigated. Surface biotin-labeling analysis revealed that the surface expression levels of integrin β1 were greatly decreased by overnight incubation with 4-AP (Figure 2A). However, co-incubation with FN-439 largely attenuated the effect of 4-AP (Figure 2A). When neurons were transfected with a plasmid expressing green fluorescent protein (GFP) and were then immunostained against integrin β1 without permeabilization, integrin β1^+^ puncta were observed in GFP^+^ dendrites; however, very few puncta were observed in 4-AP-treated neurons (Figure 2B). FN-439 largely attenuated this effect of 4-AP on integrin β1^+^ puncta (Figure 2B). Together, these results suggest that chronic elevation of network activity impairs surface expression of integrin β1.

**Figure 3:**
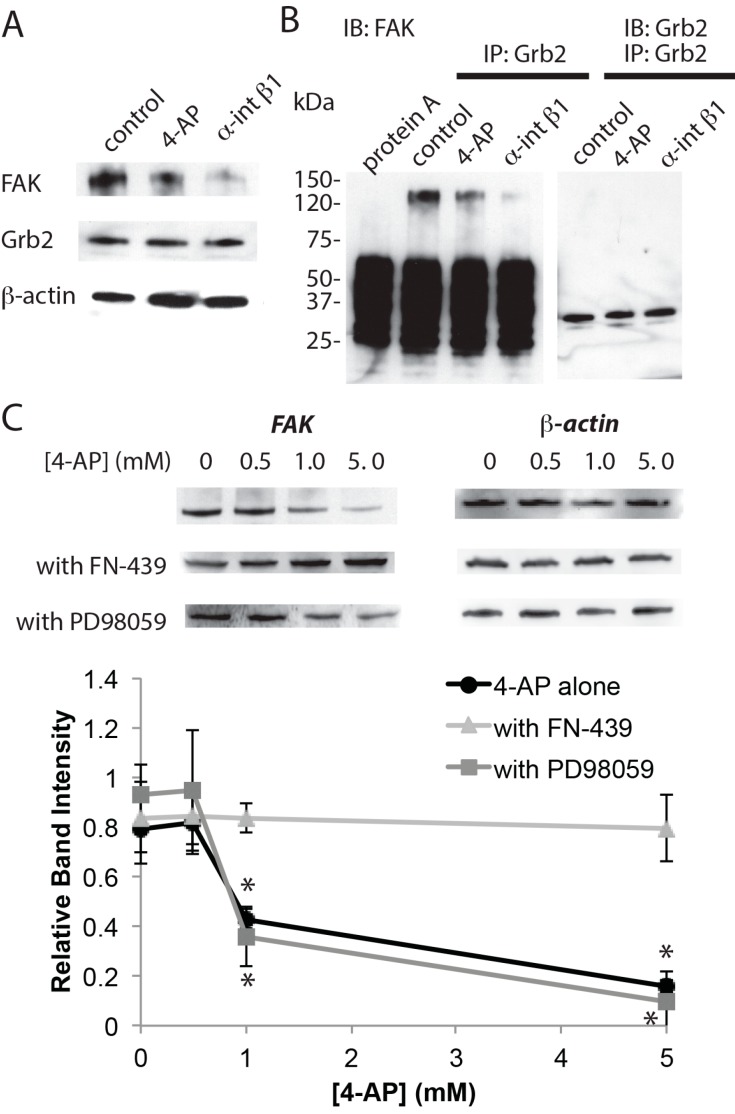
Interaction between FAK-Grb2 is impaired by chronic network activation. (**A**) Western blot analysis of FAK and Grb2; (**B**) Immuno-precipitation analysis of Grb2. Samples were immuno-precipitated with anti-Grb2 antibody, and then immuno-blotted with anti-FAK or anti-Grb2 antibodies. Neurons were incubated with 5 mM 4-AP or 50 μg/mL anti-integrin β1 antibody for 18 h; and (**C**) Inhibition of MMPs blocks the effect of chronic activation on FAK levels. Neurons were incubated with the indicated concentrations of 4-AP (with 50 μM FN-439 or 50 μM PD98059) for 18 h (*n* = 4, * *p* < 0.05, one-way ANOVA).

Then, the effect of 4-AP on FAK the downstream of integrin signaling was examined. FAK levels decreased when neurons were incubated with 4-AP, whereas levels of the adaptor protein Grb2, which interacts with FAK and activates Erk1/2 [15], were unchanged (Figure 3A). Incubation with the function-blocking anti-integrin β1 antibody also decreased FAK without changing Grb2 levels (Figure 3A). Further, immuno-precipitation with anti-Grb2 antibody also showed that levels of co-precipitated FAK were decreased by the treatment with 4-AP and also by treatment with anti-integrin β1 antibody (Figure 3B), suggesting interactions between these two proteins were impaired. When neurons were co-incubated with FN-439, FAK levels were not affected by 4-AP (Figure 3C). However, inhibiting Erk1/2 by PD98059 did not block the effect of 4-AP on FAK levels (Figure 3C). Inhibiting actin polymerase with 1 μM latrunculin A [24] resulted in significantly increased rather than decreased levels of pErk1/2 and pSer-STAT3 (340% ± 7.9% and 217% ± 14.8% of control for pErk1/2 and pSer-STAT3, respectively, *n* = 4), indicating that loss of actin filament does not cause the impairment of this signaling.

**Figure 4:**
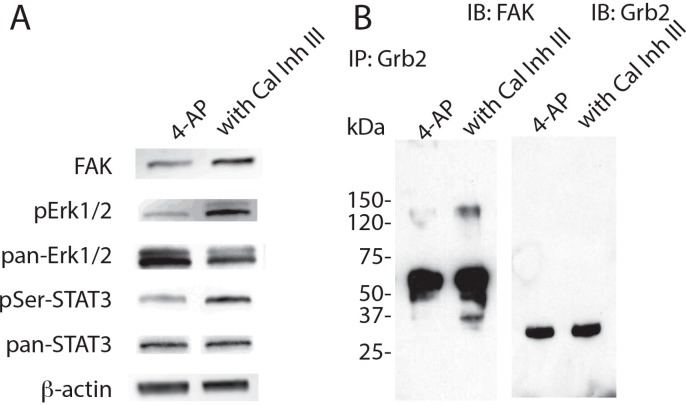
Inhibition of calpain blocks the effect of chronic activation on FAK levels. (**A**) Western blot analysis. β-Actin was used as control; and (**B**) Immuno-precipitation analysis. Samples were immuno-precipitated with anti-Grb2 antibody. Neurons were incubated with 5 mM 4-AP with or without 10 μM Cal Inh III.

To explore the mechanism by which FAK levels decrease in neurons challenged by chronic network activation, the effect of calcium-dependent proteases, calpains [25] was tested next. Calpains are known to cleave FAK upon ECM degradation [26]. Therefore it is possible that elevated neuroactivity increases intracellular calcium. Co-incubation with calpain inhibitor III (Cal Inh III) attenuated the effect of 4-AP on FAK levels, as well as total Erk1/2, pErk1/2 and pSer-STAT3 levels (Figure 4A). Immuno-precipitaion with anti-Grb2 antibody confirmed that the decrease in the levels of co-precipitated FAK was rescued by Cal Inh III (Figure 4B). These results suggest that calpain participates in the impairment of FAK-Grb2 interaction induced by chronic elevation of neuroactivity.

## 3. Discussion

In this study, the molecular events that induce vulnerability in the neurons during chronic elevation of neuroactivity were investigated. MMPs play a critical role in the impairment of Erk1/2 and STAT3 activation, severely diminishing surface expression of integrin β1. Elevated neuroactivity as well as function blocking antibody against integrin β1 caused a reduction of FAK. Immunoprecipitation experiments showed that the amount of FAK interacting with the adaptor protein Grb2 also decreased. Grb2 is known to activate Ras and its downstream kinase Erk1/2 [27,28]. Taken together, loss of FAK-Grb2 mediated Erk1/2 activation by integrin signaling is likely the cause of vulnerability in these neurons.

Elevated MMP activity by 4-AP treatment may reduce the level of ECM protein surrounding neurons. Therefore, the loss of surface expression of integrin β1 can be promoted by the loss of interaction with ECM. In addition, integrin β1 itself is a substrate for MMP [29]. Thus, both direct and indirect effects of MMP activity likely contribute to the diminished surface expression of integrin β1.

Neurons have the ability to detect net levels of input and dynamically adjust their synaptic strength to maintain appropriate activity levels. On the one hand, when such homeostatic plasticity occurs, integrin β3 has been shown to modulate levels of excitatory synaptic receptors, without altering its own surface expression levels [30]. On the other hand, integrin β1 has been implicated in a Hebbian form of plasticity, long-term potentiation [16,31,32]. However, how E/I imbalance affects integrin β1 signaling was unknown. The results presented here show that chronic elevation of activity causes a dramatic reduction of surface expression of integrin β1. This is likely the cause of the decreased level of FAK, because incubation with the function-blocking anti-integrin β1 also decreased FAK levels.

Previously, we showed that activation of Akt, another serine/threonine kinase, mediated by calcium influx through L-type channels and integrin β1 activity, is critical for neurotrophin survival signaling in neonatal neurons [33]. Interestingly, Akt activity was unaffected by the elevation of activity [10]. In fact, these neurons survived unless they were deprived with neurotrophin signaling [10]. One possibility is that neurotrophin signaling in mature neurons does not require integrin β1. Alternatively, a subpopulation of integrin β1 that engages with L-type channels is insensitive to elevations in activity.

Elevation of neuroactivity caused the marked reduction of FAK levels. This may reflect a reduction in expression level. It is also possible that post-translational modifications regulate FAK levels. It is well known that FAK undergoes cleavage mediated by caspase-3 during apoptosis [34,35]. However, induction of caspase-3 or apoptosis were not detected in the 4-AP treated neurons unless they were co-treated with the function blocking anti-TrkB antibody to block neurotrophin signaling [10]. FAK is also cleaved by calpain, a calcium dependent protease [26,36]. Since neuroactivity triggers calcium influx through the NMDA receptor and other voltage gated calcium channels, calpain is likely to be activated when neuroactivity is elevated. Indeed, the current results showed that the inhibition of calpain completely attenuated reductions of FAK by 4-AP. Although Erk1/2 has been shown to induce calpain activation [37], inhibition of Erk1/2 did not prevent degradation of FAK by 4-AP, suggesting the loss of Erk1/2 is not a cause of FAK degradation. Because the calpain cleavage site in FAK is located in the C-terminal region [38], cleavage will cause FAK to lose the domain through which it interacts with Grb2, which is also located in the C-terminal region [39,40]. The marked decrease in pErk1/2 levels by chronic elevation of excitability was observed despite the increase in total Erk1/2. Since the treatment with latrunculin A resulted in a decrease in total and an increase in phospho-Erk1/2, destabilization of actin filaments does not appear to be the direct cause. On the other hand, calpain seems to play a key role as its inhibitor reversed changes in Erk1/2 as well as FAK induced by 4-AP. Taken together, these results suggest that degradation of FAK by calpain may cause Erk1/2 impairment, although the precise impact of FAK-Grb2 interaction on Erk1/2 signaling requires further elucidation.

Elevated levels of neuroactivity such as that caused by prolonged seizures lead to excitotoxicity, directing neurons to undergo apoptosis [41]. Many neurological disorders, however, are linked to E/I imbalances without showing strong signs of excitotoxicity [4,5,6]. In these diseases, degeneration of axonal and dendritic morphology proceeds slowly, which eventually leads to the loss of neurons [7,8,9]. The molecular events that induce vulnerability in neurons during elevated neuroactivity shown in this study may provide new insights for treating patients with these diseases.

## 4. Materials and Methods

### 4.1. Reagents

FN-439 and calpain inhibitor III (Cal Inh III) were purchased from Calbiochem (La Jolla, CA, USA); 4-aminopyridine (4-AP), PD98059 and latrunculin A were purchased from Sigma-Aldrich (St. Louis, MO, USA). EZ-Link NHS-SS-Biotin (succinimidyl 2-(biotinamido)-ethyl-1,3ʹ-dithiopropionate), protein A-sepharose, and immobilized avidin were purchased from Pierce (Rockford, IL, USA).

### 4.2. Antibodies

Antibodies were used at the following dilutions: polyclonal rabbit anti-Erk1/2 antibody, polyclonal rabbit anti-phospho Erk1/2 antibody, monoclonal mouse anti-STAT3 antibody, polyclonal rabbit anti-phospho Ser-727 STAT3 antibody, polyclonal rabbit anti-FAK antibody and polyclonal rabbit Grb2 antibody (Cell Signaling Technology, Danvers, MA, USA), 1:500; monoclonal mouse anti-β-actin antibody (Sigma-Aldrich), 1:10,000; polyclonal hamster anti-integrin 1 antibody (BD Biosciences, San Jose, CA, USA), 1:200. For immunoprecipitation, polyclonal rabbit Grb2 antibody (1:100 dilution, Cell Signaling Technology) was used. For function blocking, hamster anti-integrin β1 (50 μg/mL; BD Biosciences) was used.

### 4.3. Dissociated Primary Hippocampal Culture

Culture was prepared as described previously [42]. Briefly, hippocampi from embryonic day 18 (E18) Sprague Dawley rat embryos of either sex were used for both astrocyte (plated at a density of 80,000 cells/mL) and neuron (density: 200,000 cells/mL) cultures. All experiments were carried out in accordance with the Guidelines for care and use of animals for experimental procedures by the NIH, and approved by the National Institute of Neurological Disorder and Stroke (NINDS) Animal Use and Care Committee. Astrocytes were cultured in Neurobasal (Invitrogen, Grand Island, NY, USA) with 5% fetal bovine serum (FBS, Invitrogen) in 5% CO_2_ at 37 °C for 14 days. Medium was changed completely twice weekly. Neurons were plated on confluent astrocyte beds and cultured in Neurobasal and B27 in 5% CO_2_ at 37 °C. Half of the medium was changed every 2 days. Experiments were performed between 14–21 days in vitro (DIV14 to DIV21).

### 4.4. Transfection

Transfection with 1.6 μg/mL pEGFPC1 vector (Clontech, Mountain View, CA, USA) plasmid was performed using Lipofectamine 2000 (Invitrogen) in OPTI-MEM (Invitrogen) for 30 min, then the medium was replaced with NeuroBasal Medium. Transfection was performed 4 days prior to the experiments.

### 4.5. Western Blot

Samples from dissociated culture were collected with 1× SDS loading buffer (60 μL per one 24-well culture dish). The samples were boiled for 5 min, and then applied to a 4%–10% gradient SDS gel (BioRad, Hercules, CA, USA). SDS-PAGE was performed using Tris glycine SDS buffer (KD Medical, Columbia, MD, USA). The proteins were transferred to a nitrocellulose membrane (Invitrogen) using Tris-glycine transfer buffer (Invitrogen). The membranes were blocked with 4% skim milk in phosphate buffered saline (PBS) for 30 min. Incubation with antibodies was performed in the blocking solution. Membranes were washed with Tris-buffered saline with 0.05% Tween 20. The proteins were visualized with SuperSignal West Pico System (Pierce), and detected and analyzed with a BioChemi System (UVP BioImaging Systems, Upland, CA, USA). Mean ± SEM are plotted.

### 4.6. Immunocytochemistry

Cultures were fixed with 4% paraformaldehyde without permeabilizing, and blocked with PBS containing 5% normal goat serum (NGS, Vector Laboratories, CA, USA). Primary and secondary antibodies were diluted with the blocking solution. Samples were incubated for 2 h with antibodies and washed twice for 10 min with PBS. Fluorescent images were taken with a Zeiss confocal microscope (LSM-510, Zeiss, Oberkochen, Germany) equipped with a 25× lens, and Z-stacked images from eight sections (1-μm intervals) were used.

### 4.7. Cell Surface Biotinylation

Cells were treated with the function blocking anti-integrin β1 (50 μg/mL) over night prior to surface labeling. Cells were washed two times with PBS, followed by incubation with 1 mM EZ-Link NHS-SS-Biotin on ice for 30 min. Biotinylating reagents were removed by washing three times with PBS before they were lysed on ice for 15 min with 60 μL of lysis buffer (50 mM Tris-HCl, pH 7.4, 150 mM NaCl, 1% NP-40, and protease inhibitor mixture) per well. The lysates were centrifuged at 14,000× *g* for 5 min at 4 °C, then incubated with 10% (*v*/*v*) of prewashed protein A-Sepharose or immobilized avidin for 2 h at 4 °C. The beads were washed three times with lysis buffer before the absorbed proteins were eluted by boiling for 5 min with SDS loading buffer.

### 4.8. Immuno-Precipitation Assay

Cultures were incubated for 15 min with 40 μL lysis buffer per well (150 mM NaCl, 1% NP-40 and 50 mM Tris-HCl (pH 8.0) containing a protease inhibitor cocktail (Roche), then collected and centrifuged at 12,000× *g* for 10 min. Supernatants were pre-absorbed with 10% (*v*/*v*) protein A-conjugated sepharose beads (Amersham Biosciences, Piscataway, NJ, USA) for 1 h, then centrifuged at 3000× *g* for 3 min. The supernatant was incubated with 1% (*v*/*v*) Grb2 antibody for 2 h followed by 10% (*v*/*v*) protein A-conjugated sepharose beads for 1 h. The beads were then washed with the lysis buffer twice. Proteins were eluted with 10 times (*v*/*v*) SDS sample buffer. Procedure was done at 4 °C.

### 4.9. Statistical Analyses

Statistical significance between two groups was determined with a two-tailed paired Student’s *t* test. For multiple groups, statistical comparisons were made by one-way ANOVA followed by individual group tests with the Bonferroni correction made for multiple comparisons.
